# Revealing the Proteome of Motor Cortex Derived Extracellular Vesicles Isolated from Amyotrophic Lateral Sclerosis Human Postmortem Tissues

**DOI:** 10.3390/cells9071709

**Published:** 2020-07-16

**Authors:** Natasha Vassileff, Laura J. Vella, Harinda Rajapaksha, Mitch Shambrook, Amirmohammad Nasiri Kenari, Catriona McLean, Andrew F. Hill, Lesley Cheng

**Affiliations:** 1 The Department of Biochemistry and Genetics, La Trobe Institute for Molecular Science, La Trobe University, Bundoora, VIC 3083, Australia; 18491406@students.latrobe.edu.au (N.V.); m.shambrook@latrobe.edu.au (M.S.); 18039356@students.latrobe.edu.au (A.N.K.); andrew.hill@latrobe.edu.au (A.F.H.); 2 The Florey Institute of Neuroscience and Mental Health, The University of Melbourne, Parkville, VIC 3052, Australia; 3 Department of Surgery, The University of Melbourne, The Royal Melbourne Hospital, Parkville, VIC 3050, Australia; 4 La Trobe Comprehensive Proteomics Platform, La Trobe Institute for Molecular Science, La Trobe University, Melbourne, VIC 3083, Australia; K.Rajapaksha@latrobe.edu.au; 5 Victorian Brain Bank, The Florey Institute of Neuroscience and Mental Health, The University of Melbourne, Parkville, VIC 3052, Australia; C.McLean@alfred.org.au; 6 Department of Anatomical Pathology, Alfred Health, Prahran, VIC 3181, Australia

**Keywords:** Amyotrophic Lateral Sclerosis, ALS, proteomics, exosomes, motor cortex derived extracellular vesicles, extracellular vesicles

## Abstract

Amyotrophic Lateral Sclerosis (ALS) is a neurodegenerative disease characterized by the deposition of misfolded proteins in the motor cortex and motor neurons. Although a multitude of ALS-associated mutated proteins have been identified, several have been linked to small extracellular vesicles such as exosomes involved in cell−cell communication. This study aims to determine the proteome of extracellular vesicles isolated from the motor cortex of ALS subjects and to identify novel ALS-associated deregulated proteins. Motor cortex extracellular vesicles (MCEVs) were isolated from human postmortem ALS (n = 10) and neurological control (NC, n = 5) motor cortex brain tissues and the MCEVs protein content subsequently underwent mass spectrometry analysis, allowing for a panel of ALS-associated proteins to be identified. This panel consists of 16 statistically significant differentially packaged proteins identified in the ALS MCEVs. This includes several upregulated RNA-binding proteins which were determined through pathway analysis to be associated with stress granule dynamics. The identification of these RNA-binding proteins in the ALS MCEVs suggests there may be a relationship between ALS-associated stress granules and ALS MCEV packaging, highlighting a potential role for small extracellular vesicles such as exosomes in the pathogenesis of ALS and as potential peripheral biomarkers for ALS.

## 1. Introduction

Amyotrophic Lateral Sclerosis (ALS) is a neurodegenerative disease, characterized by the deposition of ubiquitinated, aggregated proteins in Lewy body-like hyaline or skein-like inclusions in the cytoplasm of upper and lower motor neurons [1,2,3]. Worldwide, ALS affects two to three out of every 100,000 individuals [4]. Mutations in 126 genes have been implicated in ALS pathology including most recently OPTN (which encodes optineurin) and VCP (which encodes valosin-containing protein) [5,6]. However, intronic repeats in Chromosome 9 open reading frame 72 (C9ORF72) and mutations in SOD1 (which encodes superoxide dismutase 1), TARDBP (which encodes TAR DNA-binding protein 43 (TDP-43)) and FUS (which encodes fusion in sarcoma RNA-binding protein) are the most well documented [2,3,7,8,9,10,11].

ALS pathogenesis can be caused by cellular stressors, which induce the generation of stress granules, a form of cytoplasmic ribonucleoprotein particles [12,13]. These granules arise from the aggregation of nontranslating mRNAs and their RNA-binding proteins, and permit the cell to conserve energy by decreasing the translation rates of most mRNAs, with the exception of stress-responsive RNAs essential for survival [13,14]. The RNA-binding proteins associated with stress granules include Staufen1 (encoded by STAU1), a multifunctional double stranded RNA-binding protein involved in somatodendritic RNA localisation, and DExH-Box Helicase 30 (DHX30), an ATP-dependent RNA helicase which has been documented to induce stress granule formation to control global translation [15,16,17]. In ALS, TDP-43 and FUS aggregate in the cytoplasm, leading to increased stress granule formation [14,18,19,20,21,22,23]. Cellular stress has been shown to alter the composition of extracellular vesicles (EVs) secreted by cells in various systems and diseases [24,25], including ALS. [26,27]. Stress granule proteins have been shown to be packaged into extracellular vesicles such as exosomes and released into the extracellular environment [28,29].

EVs are membranous vesicles released by all cells. Small EVs (nomenclature recommended by The International Society of Extracellular Vesicles (ISEV) [30]), are characteristically 40–200 nm in size and include ‘classical’ exosomes that originate from the endosomal trafficking system [31,32,33,34]. Endocytosed vesicles fuse with early endosomes (EE) where the endocytosed material is either sorted for recycling back to the plasma membrane, via the endocytic recycling compartment, or the EE mature into late endosomes (LE) [35]. Here, the inward budding of the now multivesicular body (MVB) leads to the formation of intraluminal vesicles (ILVs) which can be loaded with cargo including proteins, RNA and DNA [32,36,37,38]. The ILVs are either degraded through MVB fusion with lysosomes, or released into the extracellular fluid upon MVB fusion with the plasma membrane, at which point they are called exosomes [39]. This biogenesis process means exosomes reflect the physiological and pathological state of the cell with events such as cellular stress and inflammation, altering exosomal content [28,29]. Exosomes can travel to target cells in proximal and distal locations, where upon recipient cell uptake they can alter cellular function [40,41,42].

Although ALS-associated misfolded TDP-43 and FUS have been documented to alter stress granule dynamics, the effect of ALS pathogenesis on exosomes in the human motor cortex has not been determined. Given exosomes are selectively packaged with cargo that reflects their parental cells’ state, the direct analysis of exosomes isolated from ALS brain tissue would enhance the current understanding of ALS pathogenesis and assist in the development of cerebrospinal fluid (CSF) or blood-based biomarkers for ALS. In this study, we enriched for small EVs, including exosomes, from human motor cortex. To keep within the nomenclature in the EV field, we have defined them as motor cortex extracellular vesicles (MCEVs). By proteomics, we show expression of stress granule proteins STAU1 and DHX30 to be enhanced in ALS MCEVs compared to neurological controls (NC). This finding not only substantiates the connection between ALS and stress granules but also alludes to the transfer of stress signals via MCEVs to target cells in ALS. We propose that STAU1, DHX30 and VCAM-1 are proteins found in MCEVs and could be developed into CSF or blood-based biomarkers for the diagnosis of ALS.

## 2. Materials and Methods

### 2.1. MCEV Isolation from Brain Tissue

ALS and NC human postmortem brain tissue samples were supplied by the Victorian Brain Bank. The entire brain was extracted from each subject within 72 h postmortem and stored at −80 °C, then a section (1–2 g) of motor cortex was removed when required. The collection and use of human postmortem brain tissue was approved by La Trobe University Human Ethics Committee (HEC18004). EVs were isolated from the human postmortem brain tissue as previously published [43]. Briefly, the frozen tissue was immediately placed on ice and sliced 2–3 mm lengthways every 100 mg. A small piece of tissue was kept as a total brain homogenate control. Eight hundred microliters of Collagenase Type 3 solution (75 U/mL of collagenase (Worthington) in Dulbecco’s phosphate-buffered saline (DPBS)) was added per 100 mg of remaining tissue prior to incubation at 37 °C in a shaking water bath. Ice cold 10× inhibition solution (5× PhosSTOP (Sigma-Aldrich; St. Louis, MO, USA), 1× cOmplete ULTRA protease inhibitor (Sigma-Aldrich; St. Louis, MO, USA), 2 mM EDTA in DPBS) was then added to a final concentration of 1× and resuspended once more. The tissue was centrifuged at 300× *g* at 4 °C for five minutes. An aliquot of the 300× *g* pellet, and whole brain homogenate control were then treated with five times their weight of 1× inhibition solution (1 mL of 10× inhibition solution, 9 mL of DPBS). The samples were homogenised using 18-, 21-, 25- and 27-gauge syringes, sonicated for 20 min and centrifuged at 10,000× *g* at 4 °C for five minutes, after which the supernatants were collected. The supernatant from the 300× *g* spin was further centrifuged at 2000× *g* at 4 °C for 10 min followed by 10,000× *g* at 4 °C for 30 min. The supernatant was overlaid on a triple sucrose cushion created by layering Fraction 4 (F4); 1 mL of 2.5 M sucrose (20 mM 4-(2-hydroxyethyl)-1-piperazineethanesulfonic acid (HEPES) in H_2_O (pH 6.4), 2.5 M protease-free sucrose, pH 7.4) with a refractive index of 1.453, followed by Fraction 3 (F3); 1.2 mL of 1.3 M sucrose (10.4 mL of 2.5 M sucrose, 9.6 mL of HEPES in H_2_O), with a refractive index between 1.3978 and 1.3958, followed by Fraction 2 (F2); 1.2 mL of 0.6 M sucrose (4.8 mL of 2.5 M sucrose, 15.2 mL of HEPES in H_2_O) with a refractive index between 1.3639 and 1.3622, in an Ultra-Clear thin-wall 13.2 mL tube (344059, Beckman Coulter; Brea, CA, USA). The gradient was centrifuged at 200,000× *g* at 4 °C for 180 min in a SW41 rotor (15U12301, Beckman Coulter; Brea, CA, USA). The fractions were subsequently collected and resuspended in ice cold DPBS prior to centrifugation at 128,000× *g* at 4 °C for 80 min in 26.3 mL polycarbonate centrifuge bottles (355618, Beckman Coulter; Brea, CA, USA) in a Type 70 Ti rotor (15U6647, Beckman Coulter; Brea, CA, USA). The pellets were collected and resuspended in 80 µl of DPBS prior to storage at −80 °C.

### 2.2. SDS-PAGE Gel Electrophoresis

Protein content was quantified and equal protein per sample was incubated in 1X lysis buffer (5 M NaCl, 1 M Tris, Triton X-100, 1% (*w*/*v*) sodium deoxycholate, 1× cOmplete ULTRA protease inhibitor) at 4 °C for 20 min before centrifugation at 2500× *g*, 25 °C for five minutes. The supernatant was extracted and mixed with 6× sodium dodecyl sulphate (SDS) loading dye before undergoing sodium dodecyl sulphate-polyacrylamide gel electrophoresis (SDS-PAGE) electrophoresis on a 4–12 % Bis-Tris Plus Gel (Invitrogen). Proteins were transferred to polyvinylidene fluoride or polyvinylidene difluoride (PVDF) or nitrocellulose membrane and probed with the desired antibody (Actin, Cell Signalling (Danvers, MA, USA) 8H10D10; Binding immunoglobulin protein (BiP)/GRP 78, BD Bioscience (San Jose, CA, USA) 610979; Calnexin, Abcam (Cambridge, UK) ab22595; DHX30, Abcam (Cambridge, UK) ab85687; Flotillin-1, BD Bioscience (San Jose, CA, USA) 610821; SOD-1, Enzo Life Sciences (Farmingdale, NY, USA) ADI-SOD-100-D; Staufen, Abcam (Cambridge, UK) ab73478; Syntenin-1, Abcam (Cambridge, UK) ab133267; TDP-43, Proteintech (Rosemont, IL, USA) 10782-2-AP; Tumor Susceptibility 101 (Tsg101), Abcam (Cambridge, UK) ab83; and VCAM-1, Abcam (Cambridge, UK) ab174279) diluted in 2.5% skim milk in PBS-T (0.05% Tween), incubated with the relevant secondary antibody (mouse IgG horseradish peroxidase (HRP) or rabbit IgG HRP) and developed with Clarity enhanced chemiluminescence (ECL) reagent (Bio-Rad; Hercules, CA, USA) as per the manufacturer’s recommendations. The membrane was imaged using ChemiDoc Touch imaging system (Bio-Rad; Hercules, CA, USA) and analysed using Image Lab 5.2.1 (Bio-Rad; Hercules, CA, USA).

### 2.3. Nanoparticle Tracking Analysis

The size of isolated vesicles was determined through Nanoparticle Tracking Analysis (NTA). The samples were diluted 1:100 filtered DPBS prior to infusion into the NanoSight NS300 (Malvern Panalytical; Malvern, UK) flow-cell through a 1 mL syringe at a flow rate of 50. Five 30-s-long videos were recorded. The NanoSight NS300 NTA software was used to analyse size and concentration of the particles.

### 2.4. Transmission Electron Microscopy

The size and morphology of exosomes was observed using Transmission Electron Microscopy (TEM). A formvar-copper-coated grid (ProSciTech; Kirwan, Australia) was glow-discharged for 60 s prior to being loaded with 6 μL of sample and incubated at 25 °C for five minutes. Excess sample was blotted off using filter paper then dabbed in 200 μL of distilled H_2_O and blotted dry on filter paper, twice. The grid was dabbed in 30 μL of uranyl acetate (Agar Scientific; Stansted, UK) for 12 s before being blot dry on filter paper. Finally, the grid was fan dried and imaged by the JEM-2100 (Jeol; Tokyo, Japan).

### 2.5. Mass Spectrometry

EV fractions were sonicated for 20 min and centrifuged at 10,000× *g*, 4 °C for five minutes. Total brain samples and EV fractions were assessed for protein concentration using a bicinchoninic acid (BCA) assay kit (Pierce; Waltham, MA, USA), as per manufacturer’s recommendations. Two micrograms of total brain samples and EV fractions were resuspended in 8 M Urea in 25 mM Tris pH = 8.3 followed by 1 µL of TCEP (tris [2-carboxyethyl] phosphine hydrochloride, 200 mM solution in water) and incubated for one hour at 21 °C. Following incubation, 4 µL of 1M IAA (iodoacetamide in water) was added and samples were incubated for 45 min at 21 °C in the dark on a ThermoMixer (Eppendorf AG; Hamburg, Germany). Eight hundred microliters of 20 mM Tris (pH 8.3) and 1 μg/µL of trypsin were added to samples and incubated overnight at 37 °C. Ten microliters of 10% trifluoroacetic acid (TFA) was then added to each sample to acidify and samples cleaned using stage-tips preparations using three plugs of Empore polystyrenedivinylbenzene (SBD-XC) copolymer disks (Sigma Aldrich; St. Louis, MO, USA) for solid phase extraction. Each procedure was as followed: washes of 50 μL methanol, 50 μL 5% acetonitrile (ACN)/0.5% TFA and elution with 50μL 85% ACN/0.5% TFA. Peptides were reconstituted in 0.1% formic acid, 2% acetonitrile and loaded onto a trap column (C_18_ PepMap 100 μm i.d. × 2 cm trapping column, Thermo Fisher Scientific) at 5 µL/minute for six minutes using Thermo Scientific UltiMate 3000 RSLCnano system and washed for six minutes before switching the precolumn in line with the analytical column (BEH C_18_, 1.7 μm, 130 Å and 75 μm ID × 25 cm, Waters). Separation of peptides was performed at 45 °C, 250 nL/minute using a linear ACN gradient of buffer A (water with 0.1% formic acid, 2% ACN) and buffer B (water with 0.1% formic acid, 80% ACN), starting from 2% buffer B to 13% B in six minutes, then 33% B over 70 min followed by 50% B at 80 min. The gradient was then increased to 95% B over five minutes and remained at 95% B for one minute. The column was then equilibrated for four minutes (water with 0.1% formic acid, 2% ACN). Data was collected on a Q Exactive HF (Thermo-Fisher Scientific; Waltham, MA, USA) in data-dependent acquisition mode using *m*/*z* 350–1500 as MS scan range at 60 000 resolution, high energy collision dissociation (HCD) peptide fragment (MS/MS) spectra were collected for the seven most intense ions per MS scan at 60 000 resolution with a normalized collision energy of 28% and an isolation window of 1.4 *m*/*z*. Dynamic exclusion parameters were: exclude isotope on, duration 30 s and peptide match preferred. Other instrument parameters for the Orbitrap were MS maximum injection time 30 ms with automatic gain control (AGC) target 3 × 10^6^, MS/MS for a maximum injection time of 110 ms with AGC target of 1 × 10^5^. The ionization method used is electrospray ionization in the positive mode.

### 2.6. Data Analysis and Protein Identification

For protein identification, raw files of the samples were searched using the Maxquant (Version 1.5.0.30). Protein sequence data for Human (v. as of 16 May 2019) downloaded from Uniprot database Common Repository of Adventitious Proteins (CRAP) used as the potential lab contaminant database. Protein identification was performed using proteomics search engine Andromeda with following parameters: variable modifications: Oxidation (M), Acetylation (Protein N-term); Fixed modifications: Carbamidomethyl (C); Digestion: Trypsin with maximum two missed cleavages; minimum peptide length for specific cleavage: seven; maximum peptide mass: 4600 Da; minimum peptide length for unspecific cleavage: eight; maximum peptide; length for unspecific cleavage: 25; false discovery rate (FDR) for peptide spectrum match (PSM) and protein: 0.01. Label-free quantification (LFQ) was done with Match between runs using a match window of 0.7 min. Large LFQ ratios were stabilized to reduce the sensitivity for outliers. Missing values from label free qualification were substituted by NA and data correlation was checked. Data normalization was done using the cyclic loess method. For differential abundance analysis, nested factorial design was setup for analysis where each subtype of disease was nested within the main disease category and contrasts for main categories were computed using linear modelling and empirical Bayes with weights.

## 3. Results

### 3.1. MCEVs Isolated Display the Characteristics of Small EVs

Vesicles were isolated from human ALS (n = 10) and NC (n = 5) postmortem motor cortex as per our previously published methods. The vesicles, isolated from fraction 2 of a density gradient, underwent a series of quality control checks to ensure absence of nonvesicle contamination and that MCEVs met minimum criteria outlined by ISEV to be classified as small EVs [34]. This was achieved through Western blot, TEM and NTA.

**Figure 1:**
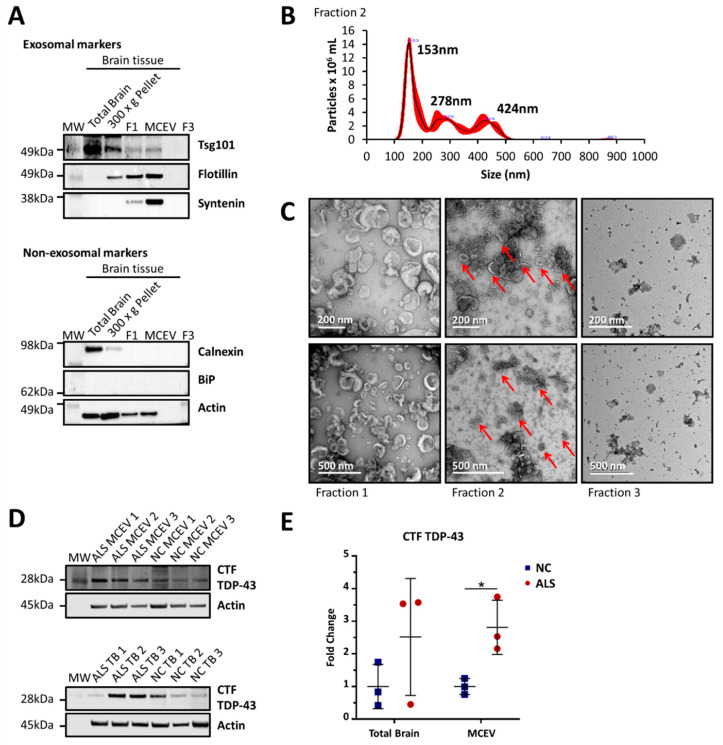
**Characterisation of motor cortex extracellular vesicles (MCEVs).** MCEVs (fraction 2) isolated from human postmortem brain tissue display the characteristics of small extracellular vesicles (EVs) resembling exosomes and are enriched in C-terminal fragment of TDP-43 (CTF TDP-43). (**A**). The vesicles in fraction 2 expressed small EV enriched markers tsg101, flotillin and syntenin, and the absence of small EV non-enriched markers; calnexin and binding immunoglobulin protein (BiP). Actin was utilized as a control. This blot is of a neurological control (NC) subject and is representative of n = 15. (**B**). Nanoparticle tracking analysis of vesicles from fraction 2 analysed by the NanoSight NS300 showed the vesicles appear to be 153 nm in diameter, a size consistent with that of small EVs such as exosomes. The peaks at 278 nm and 424 nm in fraction 2 can be attributed to the aggregation of these vesicles. This result is of a representative NC subject. (**C**). Transmission electron microscopy (TEM) images of the fractions display a population of small EVs with the size of 40–200 nm in fraction 2. Contrastingly, fraction 1 and 3 appear to contain a heterogeneous population of large EVs and small EVs, respectively. This image is of a representative NC subject. (**D**). Amyotrophic lateral sclerosis (ALS) MCEVs appear to be enriched in CTF TDP-43 compared to NC MCEVs (**E**). CTF TDP-43 expression is statistically significantly greater in ALS MCEVs compared to NC MCEVs. All protein levels were normalised to actin expression and averaged across NCs. Graphs were created using GraphPad 8. ALS n = 3 and NC n = 3, Mean ± SEM, * *p* < 0.05. Statistical significance found using Student’s t-test, with alpha = 0.05.

For Western blot analysis, the vesicular fractions (F1, F2 and F3) alongside brain homogenate lysate (Total Brain (TB) sample and 300 × g pellet, which served as cellular and enzymatic controls respectively), were probed for representative small EV enriched and small EV non-enriched markers (Figure 1A). The expression of all three small EV associated markers, flotillin-1, Tsg101 and syntenin, and absence of BiP and calnexin, suggested the presence of small EVs such as exosomes in fraction 2 in the absence of considerable cell lysis [43,44].

The identity of motor cortex vesicles in fraction 2 as small EVs was further established through NTA and TEM (Figure 1B,C). NTA analysis revealed the appearance of vesicles ~153 nm in diameter, a size consistent with small EVs such as exosomes [31,32,33,34]. NTA also revealed aggregation of vesicles as indicated by the appearance of peaks at 278 nm and 424 nm. The TEM images depicted a population of vesicles between 40 and 200 nm in diameter in fraction 2. These vesicles exhibited characteristic exosomal features such as a cup-shape morphology and a double lipid membrane [45]. From this point of the manuscript, the vesicles found in fraction 2 are referred to as MCEVs.

ALS subjects were diagnosed by the presence of TDP-43 in the frontal cortex, hippocampus and lumbar cord through immunohistochemistry by a qualified neuropathologist. NC are from postmortem subjects that did not show the presence of ALS-associated TDP-43 pathology. Upon isolation of MCEVs and TBs, we performed Western blot analysis to observe whether the 28 kDa C-terminal fragment of TDP-43 (CTF TDP-43), associated with ALS, was found in this tissue. The CTF TDP-43 was found to be at statistically significantly higher levels in ALS MCEVs compared to NC MCEVs (Figure 1D,E). The proteome composition of TBs and MCEVs was then investigated to determine whether MCEVs were selectively packaging ALS-associated proteins.

### 3.2. MCEVs Display a Unique Proteome Compared to Their respective TBs

**Figure 2:**
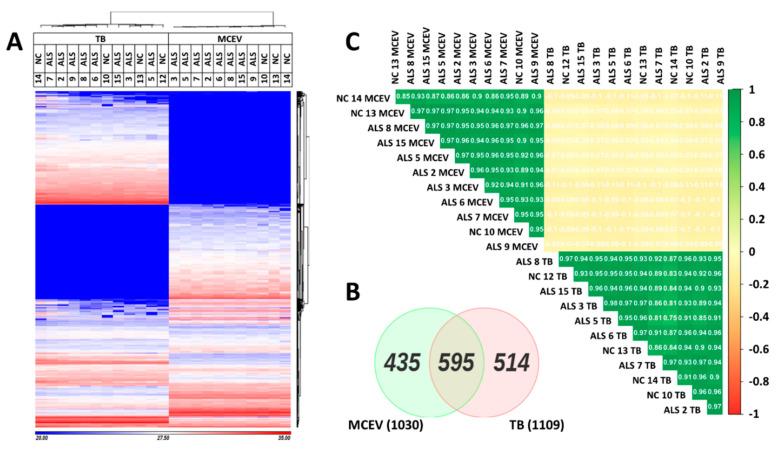
**Differential proteomic profiles of MCEVs and total brain samples (TBs) of ALS and NCs.** (**A**). Heat map depicting the relative abundancy of proteins enriched in TBs, MCEVs and common in both (label-free quantification (LFQ) values are presented in Supplementary Table S1). Protein IDs present in at least one subject across TBs and MCEVs are represented. MaxLFQ was used for analysis [46]. (**B**). Venn diagram depicting common and unique proteins identified in TBs and MCEVs. Cut off is 1 in TBs and MCEVs = present. (**C**). Correlation plot between TBs and MCEVs. The protein composition of MCEVs is similar across both ALS and NC and distinct from that of TBs. ALS TB n = 8, NC TB n = 4, ALS MCEVs n = 8 and NC MCEVs n = 3.

The proteome of the ALS and NC samples were analysed via mass spectrometry. Despite mass spectrometry analysis being performed on all 15 TB and MCEV samples, unfortunately not all the samples were successfully run or passed postrun quality control. Hence the unsuccessful samples were removed from further downstream analysis resulting in sample numbers of n = 7 for ALS MCEVs, n = 3 for NC MCEVs, n = 8 for ALS TBs and n = 4 for NC TBs. The proteome of ALS and NC MCEVs were compared to ALS and NC TBs. The results yielded distinct proteomic profiles for MCEVs and TBs, showing an enrichment of proteins in MCEVs compared to TBs, implying MCEVs may undergo selective packaging as has previously been shown (Figure 2A and Supplementary Table S1). The proteins that were observed to be abundant in both TBs and MCEVs exhibited different relative abundancy profiles between the two groups. Quantification of these abundant proteins revealed 514 proteins unique to TBs, 435 to MCEVs and 595 proteins common in both (Figure 2B). The correlation matrix demonstrated that the protein composition of MCEVs are distinct to that of TBs (Figure 2C).

### 3.3. Differentially Expressed Proteins Found in ALS MCEVs Compared to NC MCEVs

**Figure 3:**
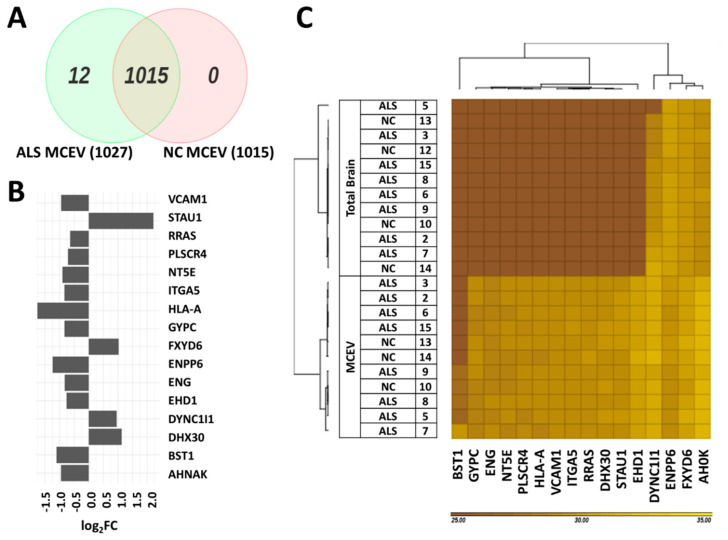
**Differentially packaged proteins between ALS and NC MCEVs.** (**A**). Venn diagram depicting number of protein IDs present in ALS MCEVs and NC MCEVs. Cut off is 2 in NC samples and 3 in ALS samples = present. (**B**). Bar graph showing statistically significant differentially packaged proteins in ALS MCEVs compared to NC MCEVs. LFQ-normalised and weighted for differential expressed analysis (MaxLFQ values are presented in Supplementary Table S2). ALS MCEVs n = 7 and NC MCEVs n = 3. (**C**). Heat map depicting relative abundancy of statistically significant enriched proteins in MCEVs compared to TBs.

**Figure 4:**
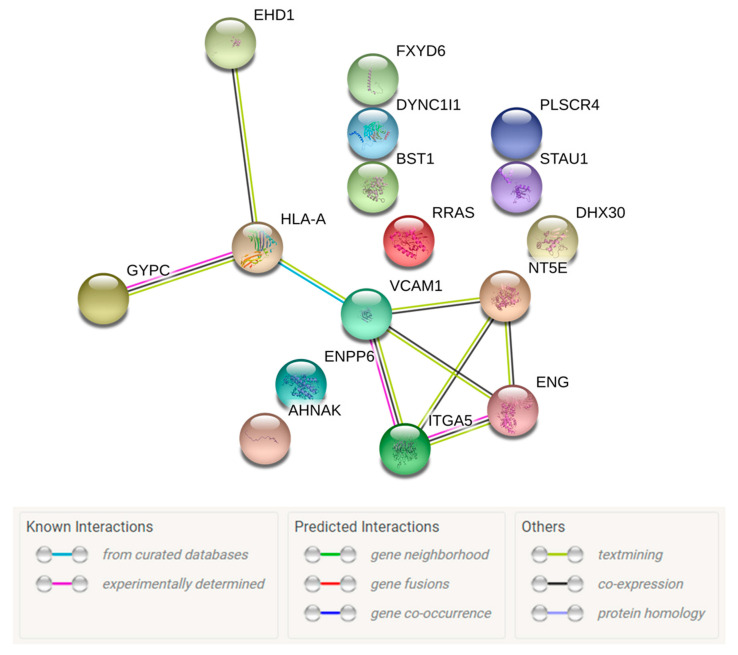
**Search tool for the retrieval of interacting genes/proteins (STRING) analysis of the 16 proteins found to be differentially packaged in ALS MCEVs compared to NC MCEVs.** Several deregulated ALS MCEV proteins appear to exhibit network relationships/connectivity. It appears GYPC and HLA-A, HLA-A and VCAM-1, VCAM-1 and ITGA5, and ITGA5 and ENG are known protein interacting partners. Graphs were created using STRING [47,48].

The mass spectrometry run detected 1027 proteins in ALS MCEVs and 1015 proteins in NC MCEVs. Although all the proteins found in NC MCEVs were also present in ALS MCEVs, there were 12 unique proteins (CD177, CHMP4B, CSPG5, DYNC1I2, IGHV3-43, LBP, RPS29, S100A9, SAA1, SCAMP4, SCN2B and SLC16A1) found only in ALS MCEVs (Figure 3A). The mass spectrometry results identified 16 proteins to be statistically significant differentially expressed between ALS and NC MCEVs however, there were no differentially expressed proteins discovered between ALS and NC TBs. The majority of these proteins appeared to mainly be downregulated in the ALS MCEVs compared to NC MCEVs (Figure 3B). Of these proteins, only STAU1, FXYD6, DYNC1I1 and DHX30 were found to be more abundant in ALS MCEVs compared to NC MCEVs (Figure 3B and Supplementary Table S2). The ALS and NC MCEVs exhibited varying relative abundance across the 16 proteins (Figure 3C). Following refinement, the 16 differentially expressed proteins underwent search tool for the retrieval of interacting genes/proteins (STRING) analysis to identify whether any of the proteins were interacting partners. Several deregulated MCEV proteins appear to exhibit network relationships, including GYPC, HLA-A, VCAM-1, ITGA5 and ENG (Figure 4). Gene ontology (GO) enrichment analysis was also performed on the 16 differentially expressed proteins to identify specific changes that may play an important role in ALS. While those enriched in ALS MCEVs compared to NC MCEVs could be used to develop future CSF and blood-based EV biomarkers. GO enrichment analysis was not performed on TB samples as there were no differentially expressed proteins observed in ALS TBs compared to NC TBs.

### 3.4. Differentially Expressed Proteins in ALS MCEVs Are Involved in Communication, Exhibit RNA-Binding Activity and Are Sequestered to Lysosomes and Exosomes

**Figure 5:**
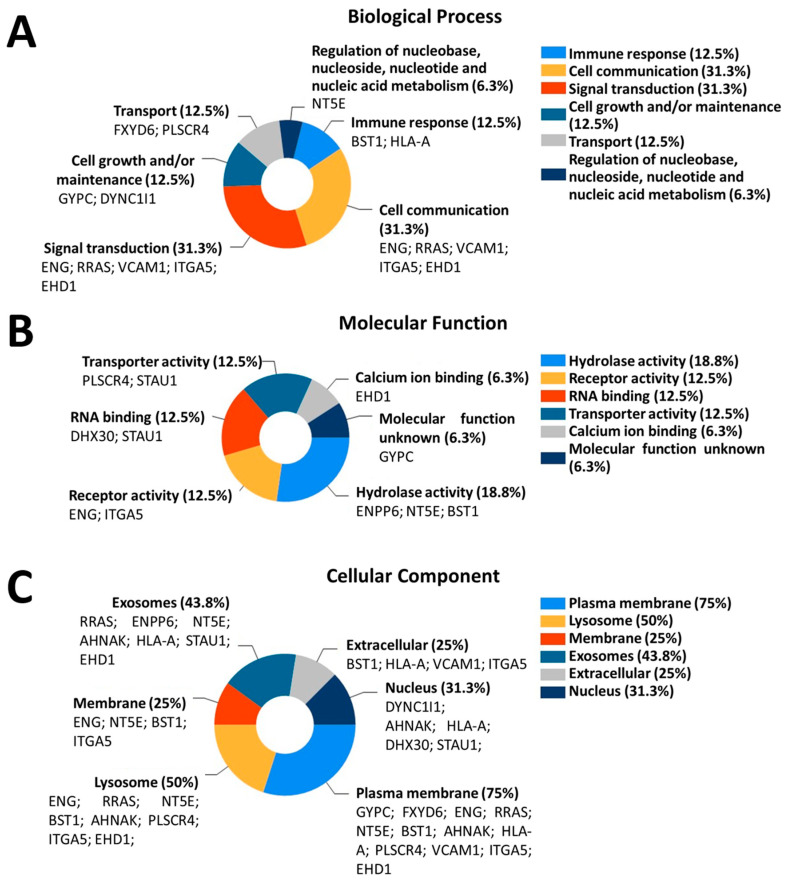
**GO enrichment of the 16 proteins found to be differentially packaged in ALS MCEVs compared to NC MCEVs.** (**A**). Biological process: The differentially expressed proteins appear to be involved mainly in cell communication and signal transduction. (**B**). Molecular function: The differentially expressed proteins appear to exhibit hydrolase activity, receptor activity, RNA-binding and transporter activity. (**C**). Cellular component: The differentially expressed proteins appear to be overwhelmingly sequestered to the plasma membrane, followed by lysosome and exosomes. Graphs were created on FunRich3.1.3 [49].

GO enrichment was performed on the 16 proteins found to be statistically significant differentially packaged in ALS MCEVs compared to NC MCEVs. Analysis of biological processes revealed the differentially expressed proteins to be mainly involved in cell communication and signal transduction, specifically VCAM-1, in addition to immune response, cell growth and/ or maintenance, transport and regulation of nucleic metabolism (Figure 5A). Molecular function analysis determined that the differentially expressed proteins exhibited hydrolase activity, receptor activity, RNA-binding activity, such as DHX30, transporter activity and calcium-binding activity. Additionally, whilst the molecular function of GYPC was found to be unknown, STAU1 was revealed to feature both RNA-binding and receptor activity (Figure 5B). Finally, upon examination of the cellular components, the differentially expressed proteins appeared to be overwhelmingly sequestered to the plasma membrane, followed by lysosome, exosomes and finally the membrane, extracellular space and nucleus (Figure 5C).

## 4. Discussion

The results of this study demonstrated enrichment of ALS MCEVs with CTF TDP-43 and revealed that these MCEVs contain 16 statistically significant differentially expressed proteins. Despite the predominant downregulation of these proteins, including those involved in cell communication, proteins with RNA-binding activity were discovered to be upregulated in ALS MCEVs. The successful isolation of MCEVs from human ALS postmortem brain tissue allowed for the study of a population of diseased EVs and their selectively packaged cargo, an observation that has not previously been achieved.

A multitude of neurodegenerative studies have documented the enrichment or selective packaging of small EVs such as exosomes with disease-associated proteins in mostly cell culture systems [41,50,51,52,53,54,55,56]. However, the selective packaging of brain-derived extracellular vesicles (BDEVs) hasn’t been well documented with few studies demonstrating the presence of disease-associated proteins in BDEVs, such as α-synuclein in dementia with lewy bodies (DLB), Aβ and phosphorylated tau in DLB and Alzheimer’s disease (AD) and toxic oligomer Aβ in AD [57,58,59]. ALS BDEVs and EVs isolated from ALS-FTD-CSF have previously been reported to be enriched in both total and 15-kDa and 28-kDa CTF TDP-43 which ‘seed’ TDP-43 aggregation in cultured cells [26,60]. Consistent with this finding was the enrichment of 28 kDa CTF TDP-43 in ALS MCEVs compared to NC MCEVs in this study. However, despite the detection of enriched TDP-43 forms in ALS MCEVs, no significant difference in phosphorylated TDP-43 (data not shown) was observed in TBs and MCEVs, an observation consistent with the literature [26]. Additionally, there is evidence associating the ALS-related mutated forms of TDP-43, SOD-1, FUS, dipeptide repeat proteins, derived from C9ORF72 intronic repeats, and increased levels of interleukin-6 with exosomes [42,61,62,63,64,65,66]. However, this is the first study revealing a panel of candidate proteins selectively packaged in MCEVs of ALS brain that may have a role in disease progression and diagnosis.

The selective packaging of ALS MCEVs uncovered an upregulation of four proteins, including STAU1, an RNA-binding protein, and DHX30, an ATP-dependent RNA helicase, which have been implicated in stress granule formation and whose RNA-binding molecular functions were successfully identified in the GO analysis [17,67,68]. The elevated levels of these proteins in ALS MCEVs, isolated from TDP-43 immunohistochemistry positive ALS brain tissues, is in accordance with the current literature, as elevated STAU1 levels have previously been detected in cells from ALS patients [15]. Additionally, STAU1 was confirmed to be an interacting partner of TDP-43 through co-immunoprecipitation and colocalisation studies, whilst DHX30 has been reported to associate with TDP-43 [67,68,69]. In addition to TDP-43, STAU1 has been determined to be a major mediator of dynein [70]. A component of which, cytoplasmic dynein 1 intermediate chain 1 (DYNC1I1), was detected to be upregulated in the ALS MCEVs. STAU1 and dynein control axonal and synaptic mRNA localisation and their dysregulation is believed to be involved in ALS related synaptic disruption [70]. Although TDP-43 has been detected in CSF exosomes through targeted proteomics [71], TDP-43 was not detected through mass spectrometry analysis in the ALS and NC TBs and MCEVs in this study. The ability of exosomes to facilitate cell-to-cell communication enables them to depict the condition of their parental cell [28,38]. Therefore, upregulation of these stress granule proteins in the MCEVs may be an indication of ALS-induced stress inflicted on parental neurons. Contrastingly, cell adhesion molecules were found to be downregulated in MCEVs.

The proteomic results revealed VCAM-1 levels to be statistically significantly downregulated in ALS MCEVs. The expression of VCAM-1 in ALS BDEVs or MCEVs has not been documented prior to this study. GO analysis identified VCAM-1 to be involved in cell communication and signal transduction, which is consistent with its role in inflammation. At the early stages of disease, upon compromise of the BBB/BSCB, inflammation is initiated, which activates cytokines resulting in upregulation of VCAM-1 in nearby neuronal cells [72,73]. However, in this study this was not observed likely because the tissue samples represent end-stage disease. Instead, the observed downregulation of VCAM-1 is supported by previous studies examining ALS tissues, which documented decreased levels of VCAM-1 in the sensorimotor cortex, cranial nuclei and spinal cord of SOD-1^G93A^ mice and an absence of elevation in VCAM-1 levels in the spinal cords of mSOD-1 mice [74,75]. These studies additionally do not detect elevated baseline levels of chemokines or cytokines, with VCAM-1 upregulation in SOD-1^G93A^ mice only occurring following injection of the cytokine IL-1β [74,75]. Furthermore, increased VCAM-1 expression has only been detected in exosomes following exposure to cytokine TNF-α [28]. Based on these findings, it appears that VCAM-1 upregulation is only initiated upon an increase in cytokine levels. Thus, given an absence of cytokines in our proteomic study, the downregulation of VCAM-1 in ALS MCEVs is consistent with the literature concerning VCAM-1 levels in an ALS tissue-specific setting.

In this proteomic study, no change in cytokine levels between ALS and NC samples was detected, possibly due to the examined tissues reflecting end-stage of disease. Furthermore, Endoglin (ENG, an accessory coreceptor for the cytokine TGF-β, Transforming growth factor beta) and Ras-related protein R-Ras (RRAS) were both found to be downregulated in ALS MCEVs. Decreased levels of ENG have previously been detected in the serum of ALS patients, this downregulation was suggested to accelerate motor neuron degeneration via the TGF-β signalling pathway [76]. Additionally, a recent study that transplanted human bone marrow endothelial progenitor cells into ALS mice, suggested the detected increase in ENG secretion by the transplanted cells was inducing BBB/BSCB endothelial cell repair in ALS [77]. Thus, the downregulation of ENG in ALS MCEVs may be indicative of disease end-stage BBB/BSCB deterioration. On the other hand, RRAS modulates ITGB1 (Integrin beta-1) activity whose upregulation, by inflammatory cytokines IL-6, TGF-β and SDF-1 (Stromal cell-derived factor-1), exacerbates disease [78,79]. The downregulation of RRAS has previously been detected in human ALS motor neurons [80], a finding consistent with that of the ALS MCEVs in this study.

Another factor released by degenerating and damaged neurons is adenosine triphosphate, which is hydrolysed by a series of nucleotide-metabolizing enzymes mainly located on microglia into adenosine [81]. 5’-nucleotidase (NT5E) is one of these enzymes, that catalyses the hydrolysis of adenosine monophosphate to adenosine [82]. The downregulation of NT5E in the ALS MCEVs may suggest there is degradation of NT5E caused by an overproduction of adenosine triphosphate by dying neurons at disease end-stage. Furthermore, ectonucleotide pyrophosphatase/phosphor-diesterase family member 6 (ENPP6) has been determined to be downregulated in SOD1^G93A^ mouse spinal cord samples and in ALS lumbar spinal cords through both gene expression and proteomic analysis [83,84], which is consistent with its expression in the ALS MCEVs. On the contrary, the upregulation of neuroblast differentiation-associated protein (AHNAK), believed to be involved in neuronal differentiation, has previously been detected in the cytosolic fraction of TDP-43 knockdown cells [85]. Given AHNAK is a known interacting partner of TDP-43 [69], the inverse relationship detected in the TDP-43 knockdown cells is consistent with the downregulated expression of AHNAK detected in the CTF TDP-43 enriched ALS MCEVs in this study. Contrastingly, phospholipid scramblase 4 (PLSCR4), involved in mediating migration of phospholipids across plasma membranes, has been found to be upregulated in the hypoglossal nucleus of SOD1^G93A^ rats [86]. Similarly, upregulation of integrin alpha-5 (ITGA5), which is involved in cell adhesion and signalling, has been detected in the spinal cords of ALS subjects [87]. The discrepancy between these studies and the results of our study may be due to the analysis of different samples types, the analysis of tissues compared to EVs or simply because the mentioned studies performed gene expression analysis whilst this study investigated protein expression levels. Despite this, the GO analysis of ALS MCEVs produced novel results that were in accordance with the current understanding of ALS.

**Figure 6:**
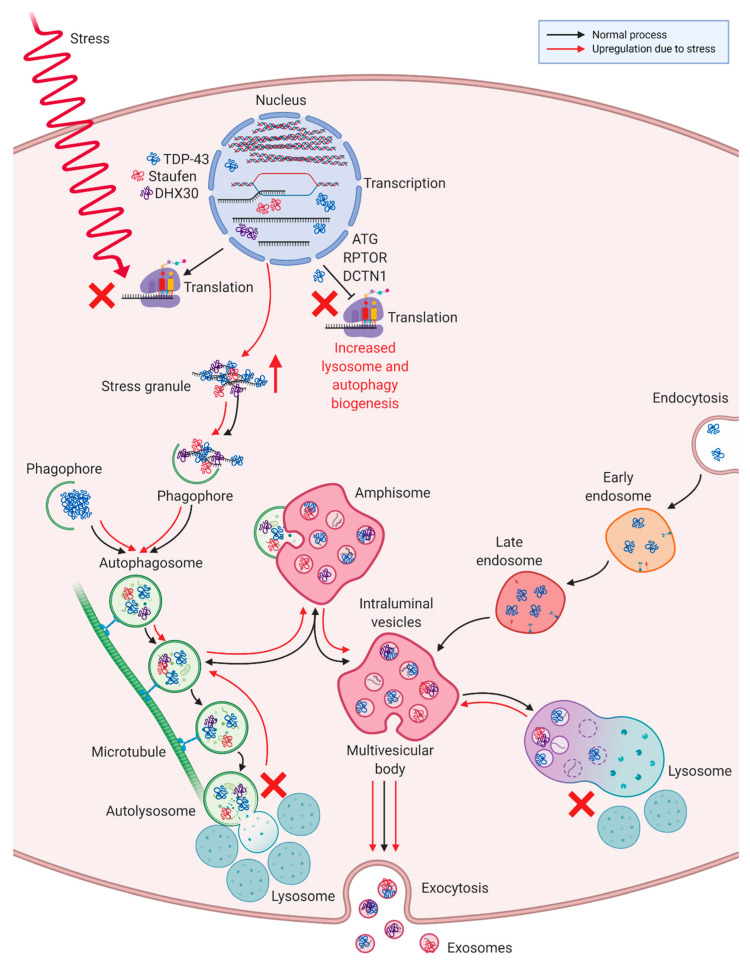
**Selective packaging of ALS MCEVs in response to ALS stress granule aggregation and lysosomal disruption. A.** Endocytosed vesicles form early endosomes (EE) which may mature into late endosomes (LE). Cargo including proteins, RNA and DNA is then transferred to the limiting membrane of the now multivesicular body (MVB) causing it to invaginate and form intraluminal vesicles (ILVs). The MVB can then fuse with lysosomes resulting in degradation of the vesicles or with the plasma membrane resulting in the release of exosomes. **B.** Macroautophagy begins with the formation of a phagophore which recognises cargo destined for degradation and surrounds it creating an autophagosome. The autophagosome is then transported down microtubules to the microtubule organising centre where it fuses with lysosomes resulting in the formation of an autolysosome and degradation of the content by lysosomal enzymes. Whilst the autophagosome is travelling down the microtubules, it can fuse and exchange material with MVBs creating an amphisome. **C.** During ALS stress, cessation of regular translation causes RNA-binding proteins such as DHX30 and Staufen1 (STAU1) to bind to mRNA and aggregate to form stress granules with which TDP-43 is known to associate. These TDP-43 positive stress granule aggregates are recognised by phagophores which initiate their degradation. However, saturation of lysosomes by aggregates, results in decreased autolysosome production and increased amphisome generation. This may result in upregulation of ILVs containing lysosomal components and TDP-43, in addition to STAU1 and DHX30 stress granule aggregates. Due to lysosomal saturation, the MVBs are obliged to fuse with the plasma membrane resulting in release of exosomes containing higher levels of lysosomal components, STAU1 and DHX30. Additionally, in ALS, TDP-43′s negative regulation of ATG7, RPTOR and DCTN1 is impeded resulting in upregulation of these genes and a consequent upregulation of lysosome and autophagy biogenesis. Image created with BioRender (biorender.com).

The 16 statistically significant differentially packaged proteins in ALS MCEVs were discovered to associate with the plasma membrane, lysosomes and exosomes, the latter of which further alludes to the exosomal identity of MCEVs. The detection of lysosomal components in ALS MCEVs alludes to an interaction with exosomal biogenesis. Lysosomes are involved in degrading cellular material sent to them through autophagy processes, such as macroautophagy, a process that sends organelles and cytosolic fractions for degradation [88,89,90]. The process of macroautophagy exhibits several similarities with exosomal biogenesis including fusion with MVBs and the employment of ESCRT proteins [88,91,92,93]. These similarities have led to the suggestion that lysosomal dysfunction may be compensated for by exosome secretion during the accumulation of misfolded proteins [94,95,96,97,98]. Thus, given the close relationship between autophagy and exosomal generation, an increase in lysosome cellular components in ALS MCEVs may be indicative of an attempt by the diseased cells to compensate for lysosomal dysfunction via loading MCEVs with certain proteins. Furthermore, TDP-43 positive stress granules and protein aggregates have been shown to be cleared by macroautophagy [70,99,100]. Inhibition of autophagy has previously been reported to significantly increase TDP-43 aggregate levels in exosomes, implicating exosomes in TDP-43 clearance and potentially in the subsequent propagation of TDP-43 proteinopathy [26]. Furthermore, autophagy markers beclin-1 and LC3-II have been documented to be upregulated following overexpression of TDP-43 CTFs [60]. Additionally, TDP-43 has been found to transcriptionally regulate proteins involved in autophagy, such as ATG7, RPTOR and DCTN1 [70,101,102]. However, the aggregation of TDP-43 impedes this resulting in upregulated lysosome and autophagy biogenesis and further TDP-43 accumulation [70,101,102]. This study reports an increase in lysosomal sequestering in addition to, an upregulation in RNA-binding stress granule associated proteins STAU1 and DHX30, in ALS MCEVs as observed by proteomics analysis. This finding suggests ALS MCEVs are undergoing selective packaging of stress granule proteins known to interact with TDP-43 [67,68,69]. This may be an attempt by the cells to counteract TDP-43 positive stress granule aggregation and the subsequent lysosomal congestion it results in following clearance through the macroautophagy pathway (Figure 6).

Given no statistically significantly differentially expressed proteins were identified between the ALS and NC TBs, GO analysis was not performed. It was surprising to discover a lack of statistically significantly differentially expressed proteins between the ALS and NC TBs. Future studies may investigate mRNA, as changes have been observed in ALS CSF small EVs and mutant SOD-1 mouse spinal cords, which were also found to differentially express exosomal and lysosomal genes [103,104]. Future studies could also investigate post translational modifications using mass spectrometry as these may reveal differences between ALS and NC in both TBs and MCEVs.

This study was the first to examine the proteomic profile of MCEVs isolated from human ALS postmortem brain tissue samples. Despite a small sample size, 16 statistically significant differentially expressed proteins were identified in ALS MCEVs compared to NCs. Further studies would involve detecting these ALS-associated proteins in CSF EVs and blood EVs to determine whether they could serve as diagnostic biomarkers. Despite these limitations, the panel of ALS MCEV proteins discovered in this study contributes to the field of EV research by providing further evidence for the selective packaging of small EVs such as exosomes. Furthermore, the identification of the RNA-binding proteins STAU1 and DHX30, in ALS MCEV suggests there may be a relationship between ALS-associated stress granules and ALS MCEV packaging, highlighting novel roles and biomarkers associated with small EVs such as exosomes in ALS.

## Supplementary Materials

The following can be found at https://www.mdpi.com/2073-4409/9/7/1709/s1: Supplementary Table S1: LFQ values of heat map displaying clustering of TB and MCEV proteins. MaxLFQ was used for analysis [46]; Supplementary Table S2: Statistically significant differentially expressed proteins in ALS MCEVs compared to NC MCEVs.
